# High-dissymmetry circularly polarized ultralong phosphorescence

**DOI:** 10.1038/s41377-023-01288-y

**Published:** 2023-09-28

**Authors:** Quan Li

**Affiliations:** Institute of Advanced Materials & School of Chemistry and Chemical Engineering, Southeast University, 211189 Nanjing, China

**Keywords:** Optical materials and structures, Photonic devices

*Advanced Materials* (2023) 10.1002/adma.202306834

Owing to the absence of appropriate helical templates, circularly polarized organic ultralong room temperature phosphorescence with a high dissymmetry factor remains a formidable challenge in contemporary research. A team of Qiang Zhao and Yanqing Lu proposes an effective tactic to enable the high dissymmetry factor by employing a circularly polarized phosphorescent system composed of meticulously designed phosphorescent polymers and self-assembled chiral helical superstructures. Specifically, multiple interactions among polymer chains can strictly restrict molecular motions of phosphorescent molecules, thereby inhibiting the non-radiative relaxation pathways and resulting in the emission decay time of 735 ms. The self-organized periodic helical superstructures serve as an ideal medium for enhancing chirality, giving rise to the attained dissymmetry factor of 1.49 and surpassing previous records by two orders of magnitude. The creatively explored system displays remarkable photo-thermal stability and demonstrates the potential applications of photoprogramming photonics. These findings would create a brilliant outlook of the optical multiplexing-based information encryption, and establish the intimate connection between circularly polarized phosphorescent materials and the evolving field of optical information technologies toward the cutting-edge photonic applications and beyond.

